# A Unique Association of Right-Sided Aortic Arch With Kommerell’s Diverticulum, Diffuse Idiopathic Pulmonary Neuroendocrine Cell Hyperplasia, and Typical Carcinoid: A Case Report

**DOI:** 10.7759/cureus.68164

**Published:** 2024-08-29

**Authors:** Adriana Nocera, Dania Nachira, Alessandra Cancellieri, Stefano Margaritora, Maria Teresa Congedo

**Affiliations:** 1 Thoracic Surgery, Fondazione Policlinico Universitario Agostino Gemelli IRCCS – Università Cattolica del Sacro Cuore, Rome, ITA; 2 Pathology, Fondazione Policlinico Universitario Agostino Gemelli IRCCS – Università Cattolica del Sacro Cuore, Rome, ITA

**Keywords:** kommerell’s diverticulum, case report, typical carcinoid, aberrant left subclavian artery, right-sided aortic arch

## Abstract

Our case presents a unique occurrence marking the first documentation of a connection between a typical carcinoid in the context of diffuse idiopathic pulmonary neuroendocrine cell hyperplasia (DIPNECH) and vascular anomalies, including a right-sided aortic arch with Kommerell’s diverticulum. Kommerell’s diverticulum is a rare congenital anomaly. The lusoria subclavian artery, another developmental anomaly, arises from the right aortic arch instead of the typical left side. Neuroendocrine cells may contribute to lung disease pathogenesis by altering their physiology before clinical symptoms appear. A 56-year-old woman with an unyielding chronic cough underwent diagnostic evaluation, unveiling rare vascular anomalies alongside a pulmonary nodule. Radiological investigations disclosed a solid nodule in the middle lobe, accompanied by proximal right-sided aortic arch ectasia and an aberrant left subclavian artery. Following multidisciplinary deliberation, thoracic and vascular surgeons elected for surgical nodule resection. Utilizing uniportal video-assisted thoracoscopic surgery, the procedure revealed the anomaly of the right-sided aortic arch. Preliminary histological examination indicated a low-grade pulmonary carcinoid, obviating the need for further lymphadenectomy due to its low malignancy potential. Subsequent histological analysis confirmed a well-differentiated neuroendocrine tumor G1 consistent with typical carcinoid within a DIPNECH framework. Currently, the patient is in follow-up. This case underscores the importance of multidisciplinary evaluation and tailored surgical approaches for managing patients with rare vascular anomalies and pulmonary nodules, emphasizing the requisite comprehensive preoperative assessment and collaborative efforts among diverse medical specialties to optimize outcomes.

## Introduction

Our case presents a unique occurrence, marking the first documentation of a connection between a typical carcinoid in the context of diffuse idiopathic pulmonary neuroendocrine cell hyperplasia (DIPNECH) and vascular anomalies, including a right-sided aortic arch with Kommerell’s diverticulum. Aguayo et al. [1] hypothesized that neuroendocrine cells may play a role in the pathogenesis of lung diseases. To support this theory, they presented six cases of patients with diffuse hyperplasia and dysplasia of pulmonary neuroendocrine cells, along with multiple carcinoid tumorlets and peribronchiolar fibrosis causing obstruction of small airways. In our case, histological examination revealed foci of DIPNECH proliferation associated with focal fibrous obliteration of some bronchioles, consistent with Aguayo-Miller syndrome.

## Case presentation

**Figure 1:**
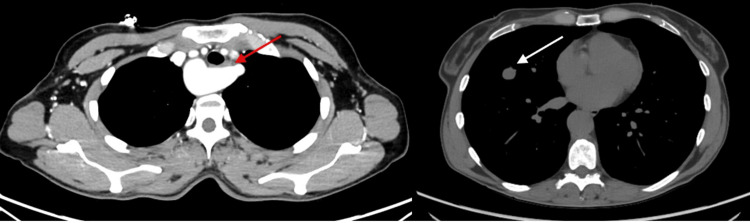
Angio-CT: right aortic arch vascular anomaly. Red arrow: the esophagus appears to be imprinted by the subclavian artery lusoria. White arrow: middle lobe nodule.

We present the case of a 56-year-old woman with a persistent chronic cough unresponsive to treatments. During the radiological diagnostic investigation, a high-resolution CT scan revealed a solid, non-calcified pulmonary nodule with sharp margins, approximately 15 mm in diameter, in the lateral segment of the middle lobe. Additionally, an ectasia of the proximal portion of the right-sided aortic arch, with a maximum diameter of 21 mm, and a left subclavian artery originating as the last supra-aortic vessel with a retro-esophageal course were noted. Subsequently, an angio-CT confirmed the right-sided aortic arch and identified the sequential origin of the supra-aortic arteries, including the left common carotid artery, right common carotid artery, right subclavian artery, and left subclavian artery. The esophagus appeared imprinted (Figure 1).

**Figure 2:**
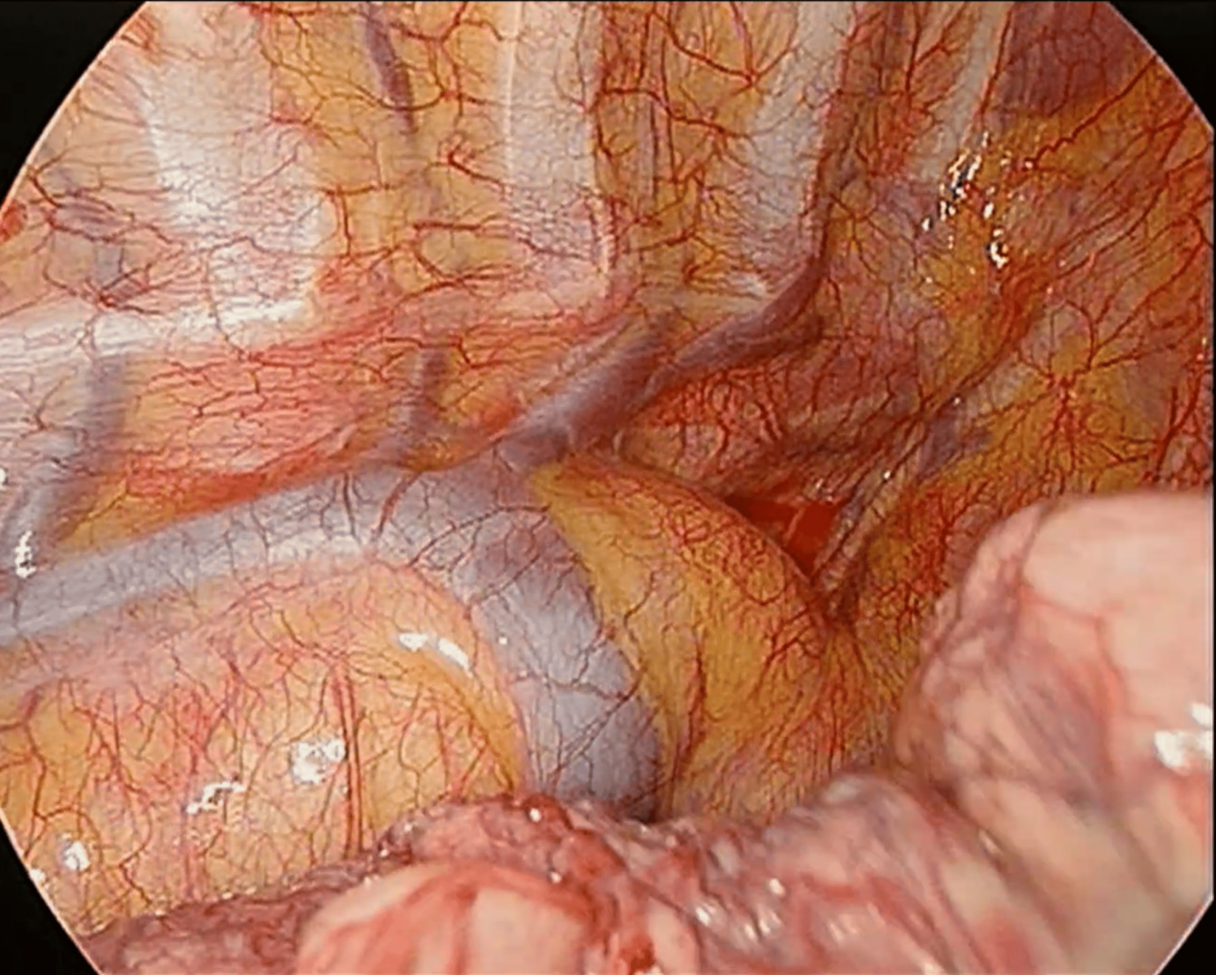
Intraoperative photo: right aortic arch and azygos vein.

In addition, the angio-CT scan revealed a homogeneous round formation in the right lung, measuring approximately 16 mm in diameter, which exhibited contrast enhancement, raising suspicion of vascular malformation. Positron emission tomography (PET) imaging showed the maximum standardized uptake value of 1.8 in the nodule in the middle lobe. The case underwent a multidisciplinary evaluation. Thoracic surgeons believed the lesion in the right lung to be solid and not a vascular malformation, as suspected in the angio-CT. As bronchoscopic characterization was not feasible due to its peripheral nature, it was decided to perform a surgical biopsy of the lesion. Vascular surgeons deemed the patient’s anatomical variant non-surgical, recommending a follow-up. During the surgical procedure, performed using uniportal video-assisted thoracoscopic surgery (U-VATS), the anomaly of the right-sided aortic arch was visualized (Figure 2).

**Figure 3:**
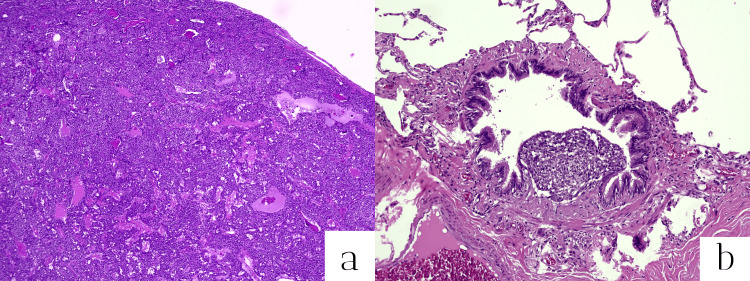
Histologic examination. (a) A well-differentiated neuroendocrine tumor (G1) of the carcinoid type of the lung associated with (B) multiple foci of proliferation, linear and polypoid, of bronchiolar neuroendocrine cells (diffuse idiopathic pulmonary neuroendocrine cell hyperplasia), associated with stenosis and focal fibrous obliteration of some bronchioles (Aguayo-Miller syndrome).

A frozen section was sent to determine the nature of the lung nodule, with a preliminary histological examination suggesting a possible low-grade epithelial neoplasm, such as a carcinoid neuroendocrine tumor (NET) G1. Therefore, it was not deemed necessary to continue the surgical procedure with a lymphadenectomy, given the low malignant potential of the lung lesion and the absence of evidence of lymph node involvement on radiological investigations such as PET. The final histological diagnosis confirmed a well-differentiated NET consistent with typical carcinoid (karyokinesis <2/2 mm^2^) within a context of DIPNECH associated with constrictive bronchiolitis (Aguayo-Miller syndrome) which could explain the long-lasting cough, unrelated to the vascular anomaly [1] (Figure 3). The vascular surgeons requested a new angio-CT scan in six months.

## Discussion

Kommerell’s diverticulum refers to the aneurysmal dilation of the descending aorta at the origin of an aberrant subclavian artery, which can occur in both the right and left aortic arches. This congenital anomaly is considered rare, with an estimated incidence of 0.05% to 0.1% in radiological series. It was first described by Kommerell, a German radiologist, in 1936 [2].

Lusoria subclavian artery is an anomaly of embryonic development where the subclavian artery originates from the right aortic arch instead of the left side, as it normally does. This condition can cause respiratory problems or swallowing difficulties if the anomalous artery presses on the esophagus or bronchi.

Three types of aortic arch diverticulum are described, including those in left-sided and right-sided aortic arches with aberrant subclavian arteries, as well as a diverticulum at the aortic-ductal junction [3]. According to the Edwards classification, a right-sided aortic arch can be categorized into the following three types: type I, with mirror image arch branches; type II, with an aberrant left subclavian artery and Kommerell’s diverticulum; and type III, with an isolated left subclavian artery communicating with the pulmonary artery [4].

Our patient presented with a right-sided aortic arch with an aberrant left subclavian artery and retro-esophageal course, along with Kommerell’s diverticulum, thus belonging to type II.

The aberrant left subclavian artery commonly courses posterior to the esophagus in 80% of cases [5]. This condition can be asymptomatic or cause symptoms due to the mass effect on the trachea and esophagus. Symptoms commonly present in adulthood, when the aortic wall becomes atherosclerotic and rigid, and include dysphagia (also known as lusoria dysphagia), cough, noisy breathing, chest pain, and lower respiratory tract infections [6].

According to Yu et al. [7], an operative indication is recommended when Kommerell’s diverticulum exceeds 1.5 times the origin of the aberrant left subclavian artery and causes obvious tracheal compression. In our case, the diameter of the aberrant left subclavian artery origin was 20 mm and Kommerell’s diverticulum did not exceed 1.5 times that measure. Moreover, the patient’s mild symptoms did not warrant surgical intervention. Indeed, the patient presented with a persistent cough unresponsive to pharmacological treatments. The pulmonary manifestation of DIPNECH, as confirmed by the final histological examination, typically presents with non-specific symptoms such as persistent cough. This suggests that the cough was due to DIPNECH rather than the vascular condition.

The main challenge would have been to perform a lymphadenectomy in this patient with vascular anomaly using the U-VATS technique. No cases of this procedure have been described in the literature in patients with this vascular anomaly and lung cancer. In our case, as it was a typical, small, peripheral carcinoid, lymphadenectomy was not mandated. Furthermore, in patients with typical forms, there is better survival compared to those with atypical forms. The choice between major resection and wedge resection does not affect overall survival [8].

Two interesting cases have been described. The first case involved a 61-year-old man with a right aortic arch and left bronchogenic carcinoma. This anatomy made mediastinal lymphadenectomy easier, given the absence of the aorta on the left side [9]. In the second case, a 56-year-old man with mid-thoracic esophageal cancer and right aortic arch, the vascular anomaly simply resulted in an approach from the opposite side during esophagectomy, the left thoracotomy approach [10]. In both cases, mediastinal lymphadenectomy was successfully performed.

Our case is unique and represents the first report in the medical literature of an association between a typical carcinoid with DIPNECH and a right-sided aortic arch with Kommerell’s diverticulum, and it stands out as the first to describe a lung surgery performed on the side of the vascular anomaly, adding another dimension to its uniqueness in the medical literature.

## Conclusions

The described case highlights the importance of a thorough preoperative evaluation and the customization of the surgical approach in patients with rare vascular anomalies, such as the right aortic arch, Kommerell’s diverticulum, and aberrant subclavian artery. These anomalies, though rare, can coexist with pulmonary conditions that produce similar symptoms, complicating the diagnosis and treatment. The involvement of a multidisciplinary team is essential to ensure comprehensive and integrated patient management. Collaboration among specialists, including the vascular surgeon, thoracic surgeon, pathologist, and radiologist, allowed for a thorough assessment of the case, customization of the surgical approach, and the provision of an accurate diagnosis.
